# Transitioning from child and adolescent psychiatry to follow-up care: a concept mapping study

**DOI:** 10.1007/s00787-026-03088-2

**Published:** 2026-06-13

**Authors:** H. C. Heek, T. Beckers, R.R.J.M. Vermeiren, E. A. Mulder, L. A. Nooteboom

**Affiliations:** 1 Department of Child and Adolescent Psychiatry, LUMC Curium, Leiden University Medical Center, Post Box 15, Leiden, 2300 AA The Netherlands; 2 Research Group Social Psychiatry and Mental Health Nursing, Hogeschool van Arnhem Nijmegen (University of Applied Sciences), Nijmegen, The Netherlands; 3 Primary Mental Healthcare, MET ggz, Roermond, The Netherlands; 4 Clinical Psychology, Behavioral Science Institute, Radboud University, Nijmegen, The Netherlands; 5 Research Institute Child Development and Education, University of Amsterdam, Nieuwe Achtergracht 127, Amsterdam, 1018 WS The Netherlands; 6 Department of Child- and Adolescent Psychiatry and Psychosocial Care, Meibergdreef 5, AmsterdamUMC, Amsterdam, 1105 AZ The Netherlands

**Keywords:** Transition of care, Child and Adolescent Psychiatry, Follow-up care, Group Concept Mapping

## Abstract

Youth with severe and enduring mental health problems often require follow-up care after treatment in child and adolescent psychiatry (CAP). In this study, follow-up care is defined as services beyond CAP, including basic mental health support, primary mental health care, and practical or parenting support delivered by community-based teams. However, transitions to such care are often fragmented, poorly coordinated, and experienced as abrupt. This study explores perspectives of CAP clinicians, follow-up care providers, parents, and youth to identify factors considered both important and feasible for improving care transitions. Using Group Concept Mapping (GCM), a participatory mixed-methods approach, participants (*n* = 55) generated, sorted, and rated statements perceived as important and feasible in transitions from CAP to follow-up care. Data were analyzed using multidimensional scaling, hierarchical cluster analysis and Go-Zone analysis. Participants generated 56 statements, thematically grouped into nine clusters: (1) Care coordination, (2) Integrated care networks, (3) Care organization, (4) Family-centered care, (5) Follow-up care, (6) Shared decision-making, (7) Handover, (8) Accessible services, and (9) Empowerment and recovery. The findings suggest that transitioning from CAP to follow-up care is multifaceted and requires more than a timely handover between care providers. Particularly, shared decision-making, recovery orientation, and family involvement emerged as central themes across clusters. Our study indicates that improving transitions may benefit from coordinated, relational, and tailored approaches that promote continuity and empower youth and families throughout the process.

**Supplementary Information:**

The online version contains supplementary material available at https://doi.org/10.1007/s00787-026-03088-2.

## Introduction

Youth experiencing severe and enduring mental health problems often require integrated support that spans multiple life domains [1, 2]. While Child and Adolescent Psychiatry (CAP) services are designed to address complex mental health needs, they are typically delivered as time-limited interventions. Nevertheless, many young people continue to require support beyond CAP, turning to other youth care services, including basic mental health support, primary mental health care, and practical and parenting support offered by community-based teams [1–4]. To ensure both continuity and quality of care, well-coordinated, timely transitions from CAP (i.e. inpatient, emergency, and outpatient psychiatric care for children and youth) to suitable follow-up care services are essential. In practice, however, these transitions are often fragmented, delayed, or insufficiently adapted to individual circumstances [5].

Effective support for youth with persistent mental health problems requires a seamless continuum that combines accessible, community-based support with specialized care such as CAP [6, 7]. Within this continuum, well-timed care transitions are essential, both from a recovery-oriented perspective and in terms of efficiency. Recovery is increasingly understood as a personal and social process that extends beyond symptom reduction. It encompasses the rebuilding of autonomy, reestablishing social connections, and re-engaging with everyday life [8–10]. Prolonged reliance on specialized services can hinder this process, fostering care dependency [8–11]. Moreover, research indicates that prolonged use of specialized services does not necessarily improve outcomes [12–14]. In contrast, timely transitions to less-intensive forms of support, such as primary or community-based services, can promote autonomy and resilience [10, 14]. Yet, in many care systems, care transitions are still more frequently oriented toward escalation than toward step-down from CAP to lower-intensity services [15–17]. This imbalance may disrupt recovery processes and continuity of care [18].

Systemic factors also drive the need for timely care transitions. Global demand for CAP services is high, and long waiting lists leave many youth and families unable to access necessary support [17, 19]. Aligning care intensity with current needs can relieve system pressures and improve access for others [19]. However, transitions from CAP to follow-up care remain challenging. Families with severe and enduring problems are typically involved with multiple professionals across diverse organizations [20]. Their problems may fluctuate in severity over time, involve multiple family members, and encounter recurring crises [1, 20–22]. In some cases, professionals perceive the overall situation “too severe” for lower-intensity services, even when clinically appropriate [23], and thus underestimate the potential for follow-up care involvement.

Organizational barriers, including long waiting lists, fragmented care pathways, and insufficient interorganizational collaborations, further complicate care transitions [20, 24]. Follow-up care is often introduced late in the care trajectory, partly due to concerns about relapse or a sense of personal responsibility among professionals [2]. Evidence from adult psychiatry suggests that professionals may lack confidence or tools to determine when someone is ready for a care transition [14]. Some professionals hesitate to engage with lower-intensity services, fearing inadequate follow-up care [14, 25]. Moreover, professionals operate within increasingly complex and continuously evolving mental healthcare systems, which may limit their overview of available and appropriate follow‑up services [26]. As a result, youth and their families may remain in specialized care longer than necessary. It remains unclear how professionals determine the timing and nature of transitions to follow-up care, and how organizational, interprofessional, and clinical factors interact in this process.

To address this gap, this study aims to identify factors considered both important and feasible in the transition from CAP to follow-up care. A Group Concept Mapping approach is employed to capture the perspectives of CAP clinicians, follow-up care providers, parents, and youth. By doing so, this study seeks to generate actionable insights that can inform the development of more continuous, recovery-oriented, and responsive care systems for youth and families living with severe and enduring mental health problems.

## Method

### Design

In our mixed methods study approach, we used Group Concept Mapping (GCM) [27] to systematically capture and structure the perspectives of care providers, parents, and youth regarding the transition from CAP to follow-up care. GCM is particularly well-suited for studying complex phenomena, as it facilitates the articulation, organization, and prioritization of tacit knowledge [27, 28]. Grounded in the principles of holistic triangulation [29], GCM enables diverse participants groups to collaboratively generate and visualize their ideas through data-driven maps [27, 28].

The Medical Ethics Review Board of Leiden University Medical Centre has determined that this research project is not subject to the Medical Research Involving Human Subject Act (WMO) and complies with the Netherlands Code of Conduct for Research Integrity (N20.200). We applied the Consolidated Criteria for Reporting Qualitative Research (COREQ), to ensure clear and comprehensive reporting of the study methods [30].

### Setting

Since 2015, the youth care system in the Netherlands has been decentralized, with municipalities carrying full organizational and financial responsibility for youth care services. This includes services ranging from prevention and early intervention to (specialized) mental health services (e.g. CAP) and child protection. In the Netherlands, mental healthcare is structured across four levels: pre-primary care (basic public services like vaccination services and general community support), primary care (including professionals such as general practitioners and community-based teams), secondary care (specialized services requiring a referral), and tertiary care (highly specialized, often CAP) [4].

Children and families with severe and enduring problems often transition between these levels of care. In the Dutch context, follow-up care from CAP is typically delivered within primary or secondary care settings. These include general practitioners (GPs), basic mental health services, basic mental health care, primary mental health workers, and community teams such as Youth and Family Teams [4]. Therefore, this study includes the perspectives of care providers operating within primary, secondary and tertiary care services, focusing specifically on the transition from CAP to follow-up care.

### Participants

Participants were recruited through purposive sampling, snowball sampling, and selective open recruitment. Recruitment for care providers took place via contacts within CAP institutions and primary and secondary care services such as community-based teams and basic mental health services. Parents and youth were recruited through parent and youth advisory boards and organizations such as the National Youth Council (NJR) and Experienced Experts (ExpEx). Additionally, a recruitment flyer was shared on LinkedIn by all authors. Interested participants who met the inclusion criteria could sign up via Google Forms, after which the researcher (HCH) contacted them.

The inclusion criteria were as follows:

- Professionals working in CAP, such as clinical child and adolescent psychologists and psychiatrists.
- Professionals working in primary and secondary care, such as community-based teams and mental health services.
- Parents with experience in the process of transitioning from CAP to follow-up care.
- Youth aged 16 and older with experience in the process of transitioning from CAP to follow-up care. We included youth aged 16 and older, as they are expected to have the necessary cognitive and evaluative skills to reflect on the process of transitioning in care, and parental consent is not required, enhancing the study’s feasibility.

Given that the recommended number of participants for GCM typically ranges between 20 and 30 [28], we aimed to include a minimum of 15 participants per group. This approach is consistent with existing methodological guidelines [31], and is supported by evidence indicating that involving 20 to 30 participants enhances the reliability and consistency of the results [32].

### Data collection and analysis

Data collection took place between February and June 2024. Specifically, the brainstorm phase ran from February to March; the sorting phase from April to May; and the ranking phase from April to June.

Before starting, participants received an information letter via email from researcher HCH and could ask questions. They then received a link to the software, reviewed the study details again, and provided informed consent, including confidentiality and withdrawal rights. Participants were informed in advance about the follow-up phases and re-invited for sorting and ranking.

We followed the phases of GCM: (1) preparation, (2) generation of statements (3) clean-up statements, (4) structuring the statements, (5) concept mapping analysis, and interpretation of the maps [27]. Data collection and analysis were conducted using Global MAX™ software (Concept Systems), which allowed participants to log in with a personal account and complete tasks at their own pace. Only the researcher could view participant names. Data were collected in Dutch and translated by the first author, with verification by the co-authors.

#### Phase 1: preparation

In this phase, the recruitment strategy, logistics, and focus prompt were established. Feedback from youth and parent experienced experts was used to optimize the clarity and relevance of the focus prompt and study materials. The focus prompt was: “A factor that I think is important when transitioning from CAP to follow-up care is…” (Dutch translation: “Een factor die volgens mij belangrijk is in het afschalen van zorg is …”).

#### Phase 2: generation of statements

During the brainstorming phase, participants were asked to respond to the prompt by listing all factors they considered important for the transition from CAP to follow-up care. They could view (anonymous) responses from others to build on each other’s input. Participants could contribute as many statements as they wished; no minimum or maximum was specified. The brainstorm phase was open for four weeks and closed once no new ideas were generated and no additional participants contributed.

#### Phase 3: clean up statements

Researchers HCH and TB cleaned the data by removing duplicates, correcting spelling and grammatical errors, separating statements with more than one factor, and merging statements with the same content but different phrasing.

#### Phase 4: structuring the statements

Participants were asked to sort the statements into meaningful clusters and named each cluster. After completing the sorting task, participants proceeded directly to ranking the statements, in which they evaluated the importance and feasibility of the mentioned factors on a 7‑point Likert scale. The questions were (translated from Dutch): (1) How important do you find this factor in the process of transitioning to follow-up care (ranging from ‘not important at all’ to ‘very important’) and (2) How feasible (practically executable) do you find this factor (ranging from ‘very feasible’ to ‘not feasible at all’).

#### Phase 5: concept mapping analysis and interpretation of the maps

This phase aimed to synthesize and interpret the data collected in phases 2–4. First, statements were mapped using multidimensional scaling (Point Cluster Map), positioning them based on how often participants grouped them together [27, 28]. Second, hierarchical cluster analysis grouped these into clusters, producing a point cluster map. A stress value was calculated to assess the fit of the data [14, 32]. We used 0.39 as the upper limit for the stress value to ensure a reliable concept mapping study [32].

In addition, pattern matches and a Go-Zone graph were generated [28]. The pattern match compared average cluster ratings across participant groups (e.g., professionals versus parents/youth), providing insight into similarities and differences in perceived importance and feasibility [28]. The Go-Zone graph plotted the perceived importance and feasibility of each statement, dividing them into four quadrants. This helped identify which factors were seen as both important and feasible, or only one of the two [28].

Furthermore, reflexivity was supported through regular reflective discussions, including team meetings with EH, RV, EM, and LN and separate meetings between EH and TB. These discussions informed the interpretation of the results and fostered reflexivity regarding the researchers’ roles and professional backgrounds (EH: doctoral researcher on integrated and transitional youth care; RV: professor and child and adolescent psychiatrist; EM: professor with expertise in qualitative research and implementation in youth care; LN: expert in integrated care and qualitative research; TB: doctoral researcher and nurse specialist in adult psychiatry). TB also contributed methodological expertise in Group Concept Mapping.

## Results

### Participants

**Fig. 1:**
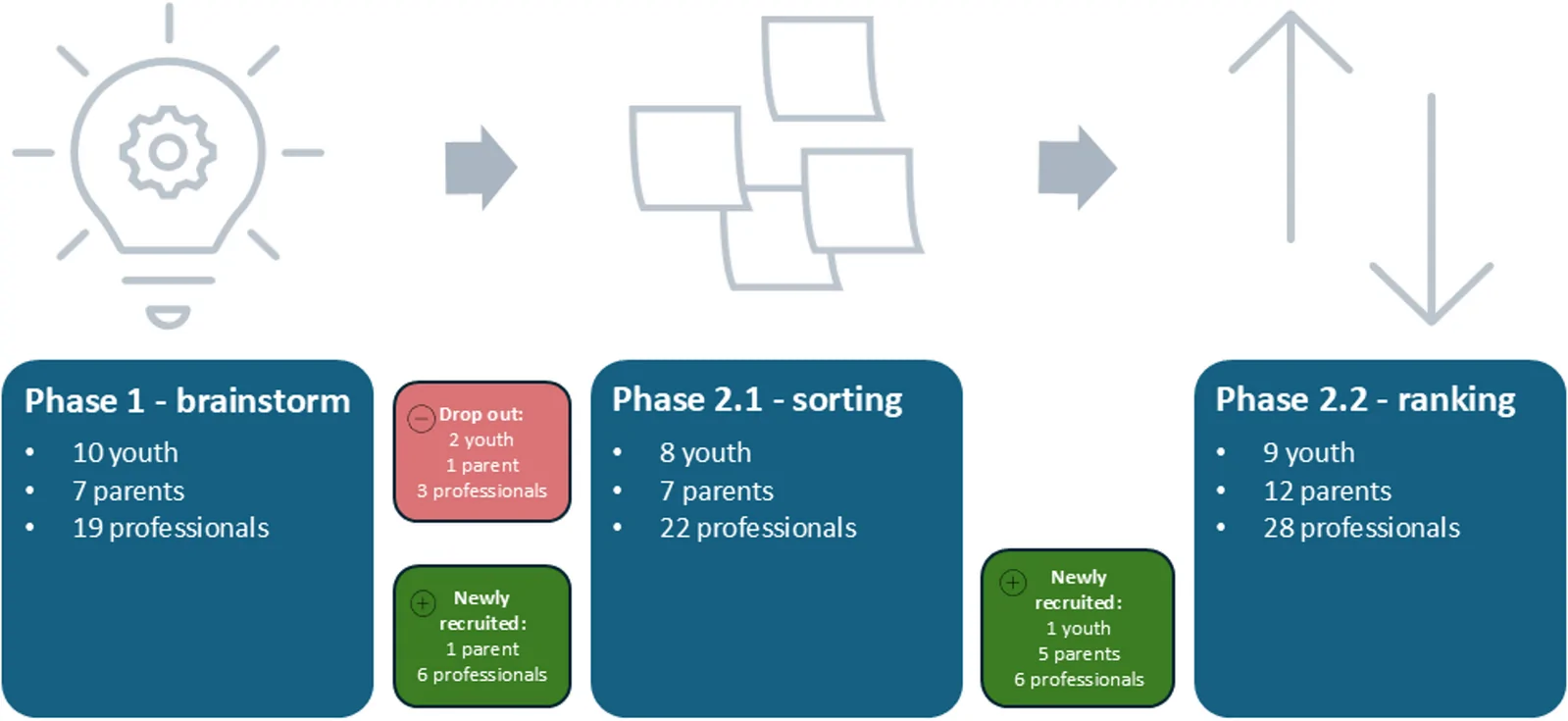
Participants per phase

As data collection took place in two phases, participant numbers varied per phase (see Fig. 1 for details). For the analysis, sorting data from 3 youth, 1 parent, and 4 professionals were excluded due to incorrect input; these participants were still included in the ranking phase. In addition, 28 professionals completed ranking question 1, but only 27 provided valid data for ranking question 2. In GCM studies, it is common to have different participants across phases and variation in responses between ranking questions [32, 33]. The study included participants from across the Netherlands, with most coming from the province of South Holland. Table 1 presents participants demographics, including care setting for professional participants (CAP or follow‑up care).

**Table 1:** Demographic information of participants by group

Characteristic	Youth (*n* = 11)	Parents (*n* = 13)	Professionals CAP (*n* = 20)	Professionals follow-up care (*n* = 11)
Mean age in years (SD)	22 (5.16)	47.69 (6.07)	40.65 (7,09)	41.91 (10.12)
Gender	Male: 1 Female: 9 Unknown: 1	Male: 2 Female: 11	Male: 2 Female: 18	Male: 1 Female: 10
Mean years of (work) experience within youth care (SD)	5.18 (2.71)	9.38 (4.23)	11.50 (5,70)	13.73 (7.27)

### Generation of statements

After 36 participants generated a total of 125 statements, data saturation was reached as additional participants contributed no new factors, or only previously mentioned factors were repeated. Following data cleaning, 56 statements remained (see Appendix B), with duplicates removed, equivalent factors consolidated, and language errors corrected.

### Concept mapping analysis and interpretation of the map

**Fig. 2:**
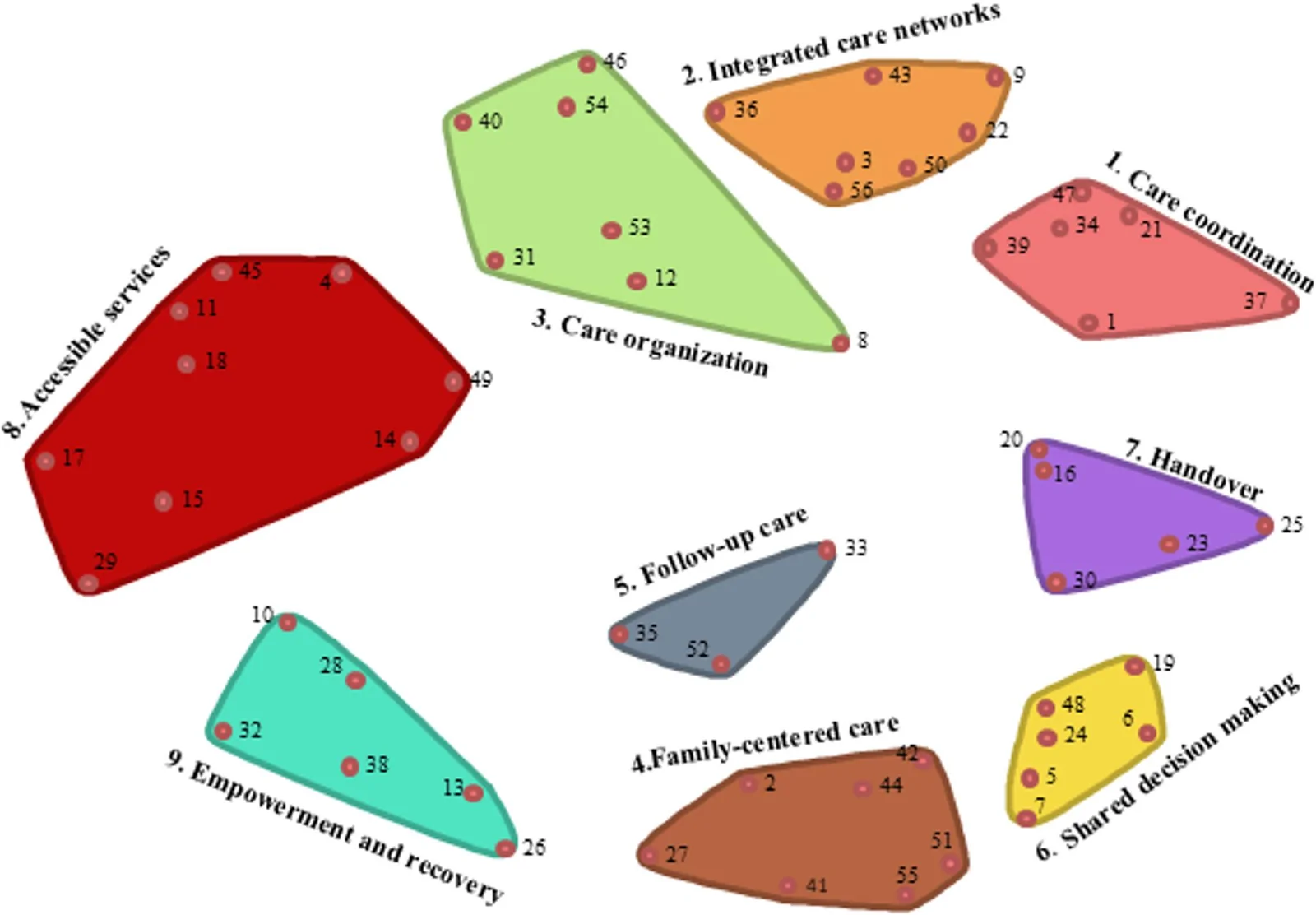
Point cluster map

The analysis revealed a stress value of 0.27, which falls below the upper threshold of 0.39 [32], indicating sufficient stability in the data to proceed with the GCM analysis. Multidimensional scaling provided a comprehensive overview of all factors mentioned by participants (see appendix A for the point map). After generating the point map, the research team (HCH, TB, EM, LN) interpreted multiple cluster map solutions to determine which configuration most accurately and meaningfully represented the data [32]. Following Rosas [33] and Beckers et al. [14], we – through iterative discussion – examined cluster maps ranging from six to fifteen clusters. This led to a nine-cluster solution, as showed in the point cluster map (see Fig. 2). Finally, the clusters were named by the research team based on participants input and cluster content

These nine clusters encompass all 56 statements and reflect key themes identified by participants as relevant to the transition from CAP to follow-up care. Below, we describe each cluster and its thematic focus.

#### Cluster 1: care coordination

This cluster includes statements related to communication, continuous involvement of care providers, and clarity regarding responsibilities during the transition process. For example: “Assign case managers who remain involved throughout the entire care process” (statement 47).

#### Cluster 2: integrated care networks

This cluster reflects the importance of collaboration between different care providers and organizations, including various forms of cooperation. For example: “More interagency collaboration: maintain involvement on a consultative basis rather than a full transfer with a new treatment trajectory” (statement 43).

#### Cluster 3: care organization

Statements in this cluster focus on the organization and structure of care, including financial aspects, the need for low-threshold and flexible consultations, and the importance of normalization as a lens through which to view the transition of youth and their families to follow-up care. For example: “Maintain a clear understanding of the local support network (the social map)” (statement 8).

#### Cluster 4: family-centered care

This cluster emphasizes alignment between treatment goals and the broader needs of families, advocating for a systemic view that integrates the perspectives of all family members. For example: “Set family-oriented goals: treatment goals should be aligned with what is “good enough” for the family within safety boundaries” (statement 51).

#### Cluster 5: follow-up care

Statements in this cluster focuses on follow-up care, highlighting what is essential in this phase, including relapse prevention planning and fostering trust and confidence in the follow-up process. For example: “Schedule evaluation moments after the care transition to monitor progress and reduce the risk of relapse” (statement 33).

#### Cluster 6: shared decision-making

Scoring highest in both importance and feasibility, this cluster contains statements about involving families in meaningful decision-making. For example: “Do not decide on transitioning care for the client, but with the client; involve the client in decisions around transitioning” (statement 6).

#### Cluster 7: handover

This cluster underlines the importance of a warm and smooth handover that is gradual rather than abrupt, as well as transparency about the options and support available after the handover. For example: “Discuss transitioning care early in the treatment process to avoid surprises; ensure clear communication and policies that recognize transitioning as an integrated part of the treatment” (statement 30).

#### Cluster 8: accessible services

As the largest cluster it addresses barriers such as waiting lists and concerns about the availability and expertise of professionals. For example: “Ensure accessible support options are available, including neighborhood teams, volunteers and mentors who can support young people or families outside the home” (statement 49).

#### Cluster 9: empowerment and recovery

This cluster centers on family empowerment and a recovery-oriented approach. For example: “Work toward confidence, self-reliance, and empowerment during treatment: strengthening both parents and children” (statement 26).

### Cluster rating and pattern match results

After selecting the best-fitting cluster configuration, we calculated the mean ratings for each cluster and statement, both in terms of importance and feasibility. An overview of all statements per cluster, along with their mean scores, is provided in Appendix C.

Overall, clusters were rated higher on importance than on feasibility. Still, the mean scores for both rankings were generally high: all clusters scored above 4.00 on a 7-point Likert scale, except statement 4 on feasibility. This indicates that participants viewed all clusters as at least moderately important and feasible.

Prior to conducting the pattern match analyses, parents and youth were examined as separate groups, as were professionals working in CAP and follow‑up care. As only minimal differences were observed within these subgroups and group sizes were limited, parents and youth were combined into one group and professionals into another. These two aggregated groups were subsequently compared using pattern matching. The results showed minimal differences (Pearson’s *r* = 0.76 for importance; *r* = 0.97 for feasibility), indicating a high level of agreement regarding the transition from CAP to follow‑up care.

### Go-Zone analysis

Next, we generated a Go-Zone, which plots all 56 statements based on their mean scores for importance and feasibility. Since the pattern matches showed little differences between participant groups, the Go-Zone combines all participants.

**Fig. 3:**
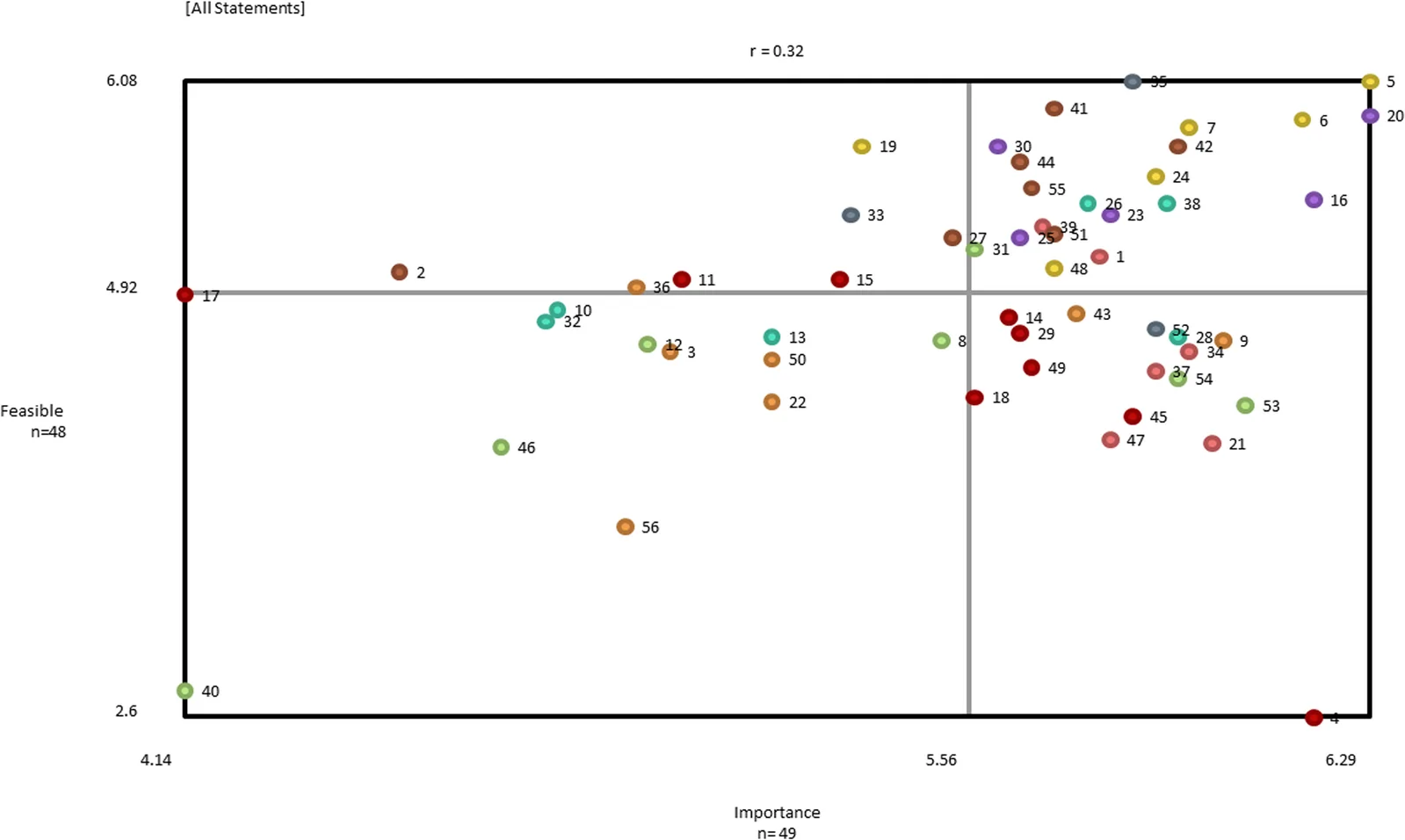
Go-Zone for importance and feasibility

The Go-Zone highlights statements rated both highly important and feasible, those in the upper-right quadrant of the graph. Each point in the graph is color-coded by cluster (see Fig. 2 for cluster colors) and numbered according to the original statements (see Appendix B). When interpreting the Go-Zone, it is important to note that the importance rankings range from 4.14 to 6.29. This means that all listed factors are generally rated as moderately to very important. For the feasibility rankings, it ranges from 2.6 to 6.08, indicating that factors are ranked from not feasible to very feasible (Fig. 3).

### Key findings from the Go-Zone

The Go-Zone analysis revealed several notable observations. First, all statements from the handover cluster fell within the Go-Zone (upper-right quadrant), with statement 20 (“Good, warm transition of care…”) rated as both the most important and most feasible factor in that cluster. This cluster includes statements emphasizing that the moment of transition should be an integrated part of the treatment process, with transparency regarding post-transition options and active involvement of both young people and their parents in the transition. In contrast, none of the factors from the ‘integrated care networks’ cluster appeared in the Go-Zone. This suggests that participants perceived these factors as either less important, less feasible, or both. Factor 5 (“Alignment with the client and parents…”) was rated highest overall on both importance and feasibility. On the other end, statement 40 (“Budget linked to the client system…”) was rated lowest on both dimensions. Statement 17 (“Investing in online support…”) was seen as fairly feasible but low in importance. Lastly, statement 4 (“Shorter or no waiting lists”) stood out as highly important but barely feasible.

## Discussion

The present study aimed to identify which factors are considered both important and feasible in the transition from Child and Adolescent Psychiatry (CAP) to follow-up care, as viewed by CAP clinicians, follow-up care providers, youth, and parents. Our findings highlight that this care transition is shaped by a wide range of factors. These were thematically grouped into nine distinct clusters, each reflecting a specific aspect of the transition process. The results suggest that a successful transition to follow-up care cannot be achieved through a single intervention or through isolated improvements. Instead, it requires coordinated attention to multiple, complementary factors. It is not merely a matter of handing over care, but involves early collaboration, continuity, and shared responsibility across services and with families.

Across clusters, importance ratings consistently exceeded feasibility ratings, suggesting a systemic gap: participants appear to have a shared understanding of what is needed for a successful transition from CAP to follow-up care, while simultaneously expressing doubts about the current system’s capacity to realize these conditions in practice. This discrepancy was most pronounced in the cluster Accessible Services (cluster 8), particularly regarding shorter or no waiting lists. Overall, the findings indicate that a holistic, recovery-oriented, and systemically integrated approach better reflects the evolving needs of youth and their families during the transition from CAP to follow-up care. In the following sections, several aspects that underpin this approach are discussed in more detail.

First, our findings indicate the need for shared commitment and engagement throughout the care transition process. This is reflected in the prominence of the ‘handover’ and ‘shared decision-making’ clusters in the Go-Zone analysis. However, recent practice-based research in the Netherlands shows that many young people in CAP receive little to no aftercare, or that the transition is abrupt and fails to consider their needs [34]. Though not the focus of the present study, other studies have highlighted that the transition from youth to adult care often occurs late and without family involvement, resulting in an unprepared and confusing experience for both youth and their families [35, 36]. Together, these findings reinforce the critical need for a deliberate and collaborative care transition process with youth and their families, regardless of the type of care being transitioned into. Future research should further explore how transitions can be shaped in partnership with youth and their families, and assess whether the Transition Navigation Model could be adapted to transitions from CAP to follow-up care within youth care services. Core features of this model, such as continuity of support, coordination across services, and active involvement of youth and families, closely align with the clusters identified in the present study. This model has shown promising results in the transition to adult mental health services, including improvements in transition readiness and a greater sense of support and engagement among youth [37]. In the Dutch context, such navigation-like roles are in principle embedded within local community teams, as also emphasized in national policy frameworks for access to local teams and integrated service delivery [38]. However, practice shows that fulfilling these coordinating and supportive roles consistently remains challenging, underscoring the need to better understand what is required to effectively operationalize such roles in everyday care without adding further complexity for youth and their families.

Secondly, this study reinforces that care transition is not merely an administrative or procedural step but a pivotal moment within the broader recovery journey. This perspective is reflected in the clusters ‘Empowerment and recovery’, ‘Family-centered care’, and ‘Care organization’, all of which emphasize the importance of supporting families in reclaiming a connection with everyday life. From this viewpoint, the shift from CAP to follow-up care becomes a moment that calls for meaning-making, trust, and continuity, rather than being treated as a logistical procedure. Recovery-oriented work with adolescents facing complex needs requires professionals to engage in “power sharing,” emotional alignment, and relational safety [39]. Young people do not recover in isolation, but through trust-based relationships in which their experiences, goals, and identities are genuinely respected. Viewed in this light, the care transition marks a relational turning point where continuity, autonomy, and agency must be actively supported. This calls for care service models that embed relational continuity into transitional care, grounded in recovery principles and tailored to the unique needs of young people and their families [40]. Future research could focus on implementation strategies to explore how these principles can be operationalized in practice and supported across organizational and professional boundaries. Furthermore, professionals need adequate training and organizational support to ensure consistent, relationship-based care across service boundaries.

This perspective is also reflected in recent research, indicating a growing movement towards recovery-oriented care that emphasizes relational continuity, participation, and inclusion [41, 42]. Care transitions are viewed as personal milestones requiring trust, connection, and active involvement of young people and their families. Fragmentation during these moments can disrupt therapeutic progress, making transparent communication and shared decision-making essential to maintaining engagement and hope throughout the care trajectory [39, 41]. Supporting recovery in this population therefore requires ongoing dialogue and continuous opportunities for youth and their families to share their stories and actively participate in decisions, not only during intake, but continuously throughout the transition to follow-up care.

Lastly, our results highlight the importance of trust, both between professionals and between professionals and families. Statements like “Pay attention to the working relationship between the client system and the professional” and “Foster trust in low-threshold forms of support” emphasize trust as a relational foundation within care transitions. Existing literature supports this, emphasizing that care transitions may challenge therapeutic relationships and risk eroding trust if not carefully managed [22, 43, 44]. For youth with enduring needs, attention to trust is especially important due to prior negative experiences with care and care transitions, which often lead to a loss of confidence in the care system [22, 45]. While our findings suggest the importance of trust, further research is needed to understand how trust can be fostered and maintained across different phases of the care transition, and how collaborative structures can support this process.

### Strengths and limitations

This study was exploratory in nature, making Group Concept Mapping (GCM) a particularly suitable method, as it is specifically designed to surface tacit knowledge [28]. A key strength is its mixed-method design, which enabled the integration of both quantitative and qualitative data to gain comprehensive insights into the transition from child and adolescent psychiatry (CAP) to follow-up care.

However, several limitations should be acknowledged. First, a ceiling effect was observed, as participants rated nearly all factors as very important and/or feasible, potentially limiting differentiation between items [46]. To address this in advance, a 7-point Likert scale was used to allow for greater response variation. Nevertheless, the limited spread in importance ratings may in part be explained by the study design: participants were asked to generate statements in response to a focus prompt that explicitly asked for factors they considered important in the context of transitioning to follow-up care. Consequently, it is to be expected that participants rated most of these self-generated items as highly important. While this reinforces the relevance of the content, it also reduced the ability to identify relative priorities. The same applies, though to a lesser extent, to the feasibility ratings, which were collected in a subsequent step.

Second, the study included only Dutch participants, ensuring relevance to the Netherlands’ healthcare system but limiting generalizability to other countries, especially low-income ones. Furthermore, the primary aim – identifying what professionals, parents, and youth consider important in the transition from CAP to follow-up care – was met with a sufficient sample size [27, 28]. However, the secondary aim of comparing more specific subgroups proved challenging due to small sample sizes within those subgroups. Consequently, youth and parents were combined into one group (families), and all professionals into another. This prevented detailed comparisons between the originally intended four groups but still allowed for the exploration of differences between families and professionals. Additionally, a notable proportion of participants dropped out, particularly between phase 1 (brainstorming) and phase 2 (sorting), likely reflecting the greater cognitive effort required by the sorting task. Several participants requested additional clarification, suggesting that task complexity may have contributed to attrition.

Third, broader social and structural factors that may influence engagement in, or access to, appropriate follow-up care did not explicitly emerge in the identified clusters. Although such factors (e.g., housing stability, financial insecurity) may play an underlying role across various aspects of the transition process, they were not articulated as distinct factors in the present study. This absence may in part be attributable to sample characteristics, as individuals with limited access to care or experiencing high psychosocial stress may be less likely to engage in research participation, potentially resulting in an underrepresentation of these perspectives in the present findings. Future research should therefore more explicitly examine how social context and access to care shape transitions from CAP to appropriate follow‑up care. In conclusion, this study demonstrates that transitions from CAP to follow-up care likely demand multifaceted, coordinated strategies emphasizing relational continuity, shared decision-making, and empowerment. These insights provide a foundation for developing recovery-oriented care pathways aligned with the complex needs of youth and families. Further in-depth research is required to better understand the rationale underlying these factors and to capture the complexity of daily practice.

## Supplementary Information

Below is the link to the electronic supplementary material.

Supplementary Material 1

Supplementary Material 2

Supplementary Material 3

## Data Availability

The datasets used during the current study are available from the corresponding author on reasonable request.

## Declarations

### AI statement

Artificial intelligence (AI) was used to support the writing process of this article. We used OpenAI’s ChatGPT (version 4) to assist with language editing, translation, and improving clarity and structure. The authors critically reviewed, edited, and approved all AI-assisted content. No AI tools were used for data collection, analysis, or interpretation. Responsibility for the content remains entirely with the authors.
