# Supplementary material for: Transitioning from child and adolescent psychiatry to follow-up care: a concept mapping study
Source: Eur Child Adolesc Psychiatry. 2026 Jun 13;35(10):3201–11. doi: 10.1007/s00787-026-03088-2 (PMC13696590; doi:10.1007/s00787-026-03088-2)
Supplement: Supplementary file 1 [file 787_2026_3088_MOESM1_ESM.docx]

Appendix C. Clusters, statements and corresponding ratings on importance and feasibility

| **Cluster (mean score for importance as scored by the participants)** | **Cluster (mean score for feasibility as scored by the participants)** | **Statement** | **Mean score of importance per statement as scored by the participants** | **Mean score of feasibility per statement as scored by the participants** |
| --- | --- | --- | --- | --- |
| 1: Care coordination (5.86) | 1: Care coordination (4.63) | 1. Timely registration with follow-up care.  21. Facilitate space for shared responsibility by improving collaboration: clear communication, breaking down silos, and reducing financial barriers.  34. Ensure joint efforts between specialized and primary care before transitioning to ensure a smooth handover.  37. Assign fixed contact persons.  39. Ensure clarity among all parties involved regarding what belongs in specialized youth mental health care, primary care, and outpatient guidance.  47. Assign case managers who remain involved throughout the entire care process. | 5.80 6.00  5.96  5.90  5.69  5.82 | 5.13  4.10  4.60  4.50  5.29  4.13 |
| 2: Integrated care networks (5.30) | 2: Integrated care networks (4.51) | 3. Do not discharge from low-threshold support services when referring to specialized youth mental health care. 9. Ensure short lines of communication and coordination with other care providers, welfare services, and schools: clear communication, task division, and easy peer consultation.  22. Ongoing involvement of specialized youth mental health care in the background: remain available for consultation and retain a "safety net" function.  36. Involve the general practitioner.  43. More interagency collaboration: maintain involvement on a consultative basis rather than a full transfer with a new treatment trajectory.  50. Establish agreements on collaboration between specialized care and informal support, such as volunteers or welfare services.  56. Assign a Youth and Family Coach to all clients from the start of care to facilitate smoother transitions from therapy to guidance-based support. | 5.02  6.02  5.20  4.96  5.76  5.20  4.94 | 4.60  4.67  4.33  4.96  4.81  4.56  3.65 |
| 3: Care organization (5.27) | 3: Care organization (4.30) | 8. Maintain a clear understanding of the local support network (the social map).  12. Support from neighborhood teams or the municipality.  31. Normalization: specialists should critically assess whether all care needs to be provided within specialized services; direct referral to general services should also be an option.  40. Link funding to the client (or client system) rather than the service provider.  46. Offer regular "day college" where primary care professionals receive expertise from specialized youth mental health professionals.  53. Provide the ability to quickly scale up in case of relapse or worsening symptoms.  54. Allow flexible consultation, advice, or short-term takeovers of treatment from specialized youth mental health care for clients with chronic psychiatric conditions. | 5.51  4.98  5.57  4.14  4.71  6.06  5.94 | 4.67  4.65  5.17  2.75  4.08  4.31  4.46 |
| 4: Family-centered care (5.54) | 4: Family-centered care (5.48) | 2. Focus less on only concrete goals and place more value on engagement.  27. Return ownership and control to the client.  41. Acknowledge and validate the client and their surroundings fears, wishes, questions, and thoughts.  42. Keep a clear view on the rationale for transitioning care: does it align with the needs and requests of the client and family? 44. Provide a positive and clear overview of goals: what has been achieved and what can still be worked on during or after the transition.  51. Set family-oriented goals: treatment goals should be aligned with what is "good enough" for the family within safety boundaries.  55. Define clear treatment goals: when is treatment considered complete, and which treatment goals have been successfully met, and which have not? | 4.53  5.53  5.71  5.94  5.65  5.71  5.67 | 5.04  5.23  5.94  5.73  5.65  5.25  5.50 |
| 5: Follow-up care (5.70) | 5: Follow-up care (5.39) | 33. Schedule evaluation moments after the care transition to monitor progress and reduce the risk of relapse.  35. Create a plan for potential relapses.  52. Foster trust in low-threshold support among youth and families. | 5.35  5.86  5.90 | 5.35  6.08  4.73 |
| 6: Shared decision-making (5.90) | 6: Shared decision-making (5.69) | 5. Align with the client and parents: what do they need (to enable transition), what are their questions, wishes, and risk factors?  6. Do not decide on transitioning care for the client, but with the client; involve the client in decisions around transitioning.  7. Continuously have a clear understanding of the client's current support needs.  19. Pay attention to the working relationship between the client system and the professional.  24. Set clear goals for specialized youth mental health care treatment: properly aligning possibilities and expectations.  48. Timing: ensure a stable moment for transitioning; assess whether the young person and family have the capacity for it at that time? | 6.29  6.16  5.96  5.37  5.90  5.71 | 6.08  5.88  5.83  5.73  5.56  5.06 |
| 7: Handover (5.91) | 7: Handover (5.53) | 16. A phased transition approach: avoid abrupt discontinuation and prevent sudden shifts from highly intensive to nearly no support.  20. Ensure a warm handover of care: open and transparent, involving parents and youth, setting clear expectations, and ensuring clarity about therapy, medication, etc.  23. Ensure clarity about the available options for transitioning care.  25. Clarify who holds responsibility at each stage and how much autonomy the young person has at different phases.  30. Discuss transitioning care early in the treatment process to avoid surprises; ensure clear communication and policies that recognize transitioning as an integrated part of the treatment. | 6.18  6.29  5.82  5.65  5.61 | 5.44  5.90  5.35  5.23  5.73 |
| 8: Accessible services (5.45) | 8: Accessible services (4.46) | 4. Reduced or no waiting lists.  11. Involve experts by experience.  14. Provide an aftercare program: offering support in reintegration and participation in society and ensuring clarity about what the aftercare program entails.  15. Consider not only focusing individual support but also on collective (social) opportunities.  17. Invest in online support that can be accessed as a fallback option.  18. Ensure adequate knowledge, skills, and expertise in low-threshold care.  29. Provide support for reintegration and participation in society.  45. Ensure stability in primary care: guarantee the continuity of support services for children and families with complex problems.  49. Ensure accessible support options are available, including neighborhood teams, volunteers and mentors who can support young people or families outside the home. | 6.18  5.04  5.63  5.33  4.14  5.57  5.65  5.86  5.67 | 2.60  5.00  4.79  5.00  4.92  4.35  4.71  4.25  4.52 |
| 9: Empowerment and recovery (5.41) | 9: Empowerment and recovery (4.97) | 10. Improve screening procedures at intake.  13. Sufficient symptom reduction  26. Work toward confidence, self-reliance, and empowerment during treatment: strengthening both parents and children.  28. Build a support network and safety net.  32. Critically evaluate the need to retain diagnoses and classifications.  38. Support practicing learned skills in the client’s own environment. | 4.82  5.20  5.78  5.94  4.80  5.92 | 4.83  4.69  5.42  4.69  4.77  5.42 |
