# Supplementary material for: Transitioning from child and adolescent psychiatry to follow-up care: a concept mapping study
Source: Eur Child Adolesc Psychiatry. 2026 Jun 13;35(10):3201–11. doi: 10.1007/s00787-026-03088-2 (PMC13696590; doi:10.1007/s00787-026-03088-2)
Supplement: Supplementary file 2 [file 787_2026_3088_MOESM2_ESM.docx]

Appendix B. Statements generated

| # | **Statement (statement prompt: “A factor that I think is important when transitioning from CAP to follow-up care is ...”** |
| --- | --- |
| 1 | Timely registration with follow-up care. |
| 2 | Focus less on only concrete goals and place more value on engagement. |
| 3 | Do not discharge from low-threshold support services when referring to specialized youth mental health care. |
| 4 | Reduced or no waiting lists. |
| 5 | Align with the client and parents: what do they need (to enable transition), what are their questions, wishes, and risk factors? |
| 6 | Do not decide on transitioning care for the client, but with the client; involve the client in decisions around transitioning. |
| 7 | Continuously have a clear understanding of the client's current support needs. |
| 8 | Maintain a clear understanding of the local support networks (the social map). |
| 9 | Ensure short lines of communication and coordination with other care providers, welfare services, and schools: clear communication, task division, and easy peer consultation. |
| 10 | Improve screening procedures at intake. |
| 11 | Involve experts by experience. |
| 12 | Support from neighborhood teams or the municipality. |
| 13 | Sufficient symptom reduction. |
| 14 | Provide an aftercare program: offering support in reintegration and participation in society and ensuring clarity about what the aftercare program entails. |
| 15 | Consider not only individual support but also collective (social) opportunities. |
| 16 | A phased transition approach: avoid abrupt discontinuation and prevent sudden shifts from highly intensive to nearly no support. |
| 17 | Invest in online support that can be accessed as a fallback option. |
| 18 | Ensure adequate knowledge, skills, and expertise in low-threshold care. |
| 19 | Pay attention to the working relationship between the client system and the professional. |
| 20 | Ensure a warm handover of care: open and transparent, involving parents and youth, setting clear expectations, and ensuring clarity about therapy, medication, etc. |
| 21 | Facilitate space for shared responsibility by improving collaboration: clear communication, breaking down silos, and reducing financial barriers. |
| 22 | Ongoing involvement of specialized youth mental health care in the background: remain available for consultation and retain a "safety net" function. |
| 23 | Ensure clarity about the available options for transitioning care. |
| 24 | Set clear goals for specialized youth mental health care treatment: properly aligning possibilities and expectations. |
| 25 | Clarify who holds responsibility at each stage and how much autonomy the young person has at different phases. |
| 26 | Work toward confidence, self-reliance, and empowerment during treatment: strengthening both parents and children. |
| 27 | Return ownership and control to the client. |
| 28 | Build a support network and safety net. |
| 29 | Provide support for reintegration and participation in society. |
| 30 | Discuss transitioning care early in the treatment process to avoid surprises; ensure clear communication and policies that recognize transitioning as an integrated part of the treatment. |
| 31 | Normalization: specialists should critically assess whether all care needs to be provided within specialized services; direct referral to general services should also be an option. |
| 32 | Critically evaluate the need to retain of diagnoses and classifications. |
| 33 | Schedule evaluation moments after the care transition to monitor progress and reduce the risk of relapse. |
| 34 | Ensure joint efforts between specialized and primary care before transitioning to ensure a smooth handover. |
| 35 | Create a plan for potential relapses. |
| 36 | Involve the general practitioner. |
| 37 | Assign fixed contact persons. |
| 38 | Support practicing learned skills in the client’s own environment. |
| 39 | Ensure clarity among all parties involved regarding what belongs in specialized youth mental health care, primary care, and outpatient guidance. |
| 40 | Link funding to the client (or client system) rather than the service provider. |
| 41 | Acknowledge and validate the client and their surroundings fears, wishes, questions, and thoughts. |
| 42 | Keep a clear view on the rationale for transitioning care: does it align with the needs and requests of the client and family? |
| 43 | Improve interagency collaboration: maintain involvement on a consultative basis rather than a full transfer with a new treatment trajectory. |
| 44 | Provide a positive and clear overview of goals: what has been achieved and what can still be worked on during or after the transition. |
| 45 | Ensure stability in primary care: guarantee the continuity of support services for children and families with complex problems. |
| 46 | Offer regular "day college" where primary care professionals receive expertise from specialized youth mental health professionals. |
| 47 | Assign case managers who remain involved throughout the entire care process. |
| 48 | Timing: ensure a stable moment for transitioning; assess whether the young person and family have the capacity for it at that time? |
| 49 | Ensure accessible support options are available, including neighborhood teams, volunteers and mentors who can support young people or families outside the home. |
| 50 | Establish agreements on collaboration between specialized care and informal support, such as volunteers or welfare services. |
| 51 | Set family-oriented goals: treatment goals should be aligned with what is "good enough" for the family within safety boundaries. |
| 52 | Foster trust in low-threshold support among youth and families. |
| 53 | Provide the ability to quickly scale up in case of relapse or worsening symptoms. |
| 54 | Allow flexible consultation, advice, or short-term takeovers of treatment from specialized youth mental health care for clients with chronic psychiatric conditions. |
| 55 | Define clear treatment goals: when is treatment considered complete, and which treatment goals have been successfully met, and which have not? |
| 56 | Assign a Youth and Family Coach to all clients from the start of care to facilitate smoother transitions from therapy to guidance-based support. |
