# Supplementary material for: Transitioning from child and adolescent psychiatry to follow-up care: a concept mapping study
Source: Eur Child Adolesc Psychiatry. 2026 Jun 13;35(10):3201–11. doi: 10.1007/s00787-026-03088-2 (PMC13696590; doi:10.1007/s00787-026-03088-2)
Supplement: Supplementary file 3 [file 787_2026_3088_MOESM3_ESM.docx]

Appendix A: point map

***** The proximity of statement points reflects how often participants grouped them together, with closer points indicating a higher likelihood of being placed in the same cluster.
